# Environmental Regulation Competition and Carbon Emissions

**DOI:** 10.3390/ijerph20010736

**Published:** 2022-12-30

**Authors:** Lizhi Cui, Yining Ding, Xiangqian Li

**Affiliations:** 1 School of Business, Anhui University of Technology, Ma’anshan 243032, China; 2 Anhui Institute of Innovation-Driven Development, Ma’anshan 243032, China

**Keywords:** carbon emissions, environmental regulatory competition, evolutionary game, spatial Durbin model

## Abstract

To clarify the relationship between environmental regulatory competition and carbon emissions and provide a theoretical basis for carbon emission reduction governance, this paper explores the strategic interaction behavior of environmental regulatory competition by constructing a three-way evolutionary game model based on the perspective of the fusion of environmental federalism and local government competition theory. On this basis, the specific forms of carbon emission reduction competition are tested using the spatial Durbin model, and the mechanism of the effect of environmental regulation competition on carbon emissions is analyzed. The evolutionary game model shows that local governments make strategic choices based on the costs and benefits of environmental regulation, and there are strategic equilibria of “race to the bottom”, “race to the top”, and “differentiation of competition”. The empirical results show that the competition for environmental regulations as a whole after the 18th National Congress of the Communist Party of China is a “race to the top”, and the increase in the intensity of environmental regulations has an inhibitory effect on carbon emissions, which remains valid after a series of robustness tests. There is heterogeneity in environmental regulatory competition, and the effect of emissions reduction is most obvious in the central region. Mechanism analysis shows that environmental regulatory competition affects carbon emissions mainly through the effect of political performance assessment, the effect of industrial structure optimization, and the effect of low-carbon technology capability improvement. Therefore, the central government should follow the local government interest function and balance the interests of all parties, appropriately increase the proportion of environmental performance assessment and optimize the performance assessment system, and consider regional development differences to find the right carbon emissions reduction path.

## 1. Introduction

With accelerated industrialization and rapid economic development in various regions of China, China’s energy demand has grown rapidly. From 2003 to 2019, China’s energy consumption increased from 1.97 billion tons of standard coal to 4.86 billion tons of standard coal, an increase of 146.7%. Fossil energy combustion is not only one of the main sources of atmospheric pollutants but also the most important source of greenhouse gas emissions. China must take strict energy conservation and emission reduction measures to face serious energy problems and environmental issues. Among them, policy formulation by the central government and policy implementation by local governments are the key to the effectiveness of the regulatory policy, but there are interactions between the central government and local governments, as well as behavioral strategies between local governments, i.e., environmental regulation competition, and how to make local governments follow the central government’s policy in environmental policy implementation and fully exert the effectiveness of environmental regulation policy is a problem that needs attention.

China has made long-term plans and efforts to reduce carbon emissions. The Twelfth Five-Year Plan has, for the first time, proposed binding targets for carbon emissions reduction and “implemented a veto system for environmental protection,” indicating that local officials will only be promoted if they meet the specified emission reduction targets or if their performance meets the emission reduction requirements. The report of the 18th National People’s Congress (NPC) incorporated the construction of ecological civilization into the Five-Sphere Integrated Plan along with political, economic, social, and cultural construction and proposed the concept of “Beautiful China” from the national strategic level. The report of the 19th NPC named pollution prevention and control as one of the three critical battles, focusing on solving the outstanding environmental pollution problems. In September 2020, President Xi Jinping proposed at the 75th United Nations General Assembly that “China’s CO_2_ emissions strive to peak by 2030 and work towards achieving carbon neutrality by 2060”. Subsequently, relevant ministries and commissions had made specific plans and detailed designs for achieving the goals of “carbon peaking” and “carbon neutrality” on several important occasions, and “carbon peaking” and “carbon neutrality” were incorporated into the overall layout of ecological civilization construction. The report of the 20th NPC pointed out that we should actively and steadily promote carbon neutralization, focus on controlling fossil energy consumption, and gradually shift to a “dual control” system for total carbon emissions and intensity. In the context of stronger environmental performance assessment, will the competition for environmental regulation among local governments remain the same or change? What impact will this have on carbon emissions?

In the vertical setting of China’s eco-environmental governance system, environmental protection responsibilities are mainly divided according to administrative regions, with the central government being responsible for the formulation of environmental protection policies and their assessment indicators, while local governments are responsible for environmental protection and are the main implementers of environmental protection policies within their jurisdictions. The separation of central governments from those who implement environmental protection policies is not only prone to information distortion but also gives local governments more authority and discretion in implementing environmental regulations. Therefore, the real attitude of local governments toward environmental regulation and their behavioral choices in implementing it directly affects whether environmental protection policies can achieve their intended goals. Existing studies have shown that when driven by economic interests, local governments tend to relax environmental regulations to gain a competitive advantage over neighboring governments when faced with competition for mobility factor resources such as capital and technology [1,2]. With the trend of gradually strengthening environmental performance assessment, it is worth exploring whether the strategic choice of local governments to carry out environmental management is “healthy competition”. Under the vision of “carbon peak” and “carbon neutral”, the impact of environmental regulation competition among local governments on carbon emissions also needs to be further analyzed. The purpose of this paper is to use an evolutionary game model to theoretically analyze the forms of environmental regulatory interactions of local governments for carbon emission reduction and to analyze environmental regulation competition and its impact on carbon emission empirically by using the spatial Durbin model to provide a theoretical basis for reducing carbon emissions and realizing green development under China’s environmental management system.

The rest of the paper is structured as follows. Section 2 presents a literature review on the interaction of local government environmental regulation strategies and the effect of environmental regulation on carbon emissions, and Section 3 provides a theoretical analysis and hypotheses on the form of strategic interaction of environmental regulation on carbon emissions. The empirical model and data description are given in Section 4. Section 5 presents the analysis of empirical results, including heterogeneity analysis and mechanism analysis. Section 6 presents some conclusions.

## 2. Literature Review

### 2.1. Environmental Regulation of Competition

Current research on environmental regulatory competition focuses on three aspects: relocation of firms, types of environmental regulatory competition, and the impact of environmental regulatory competition. The first aspect of this study focuses on the private sector’s response to regional differences in the stringency of environmental regulations from the perspective of corporate investment decisions [3]. Economic investments tend to move to jurisdictions with less stringent environmental regulations, thus indirectly indicating the existence of environmental regulatory competition. However, some literature suggests that environmental regulatory competition involves the behavior of governments rather than enterprises [1,4], and it is difficult to justify environmental regulatory competition if no private sector investment relocation behavior is observed. What is more, when Potoski investigated the U.S. air pollution control program [5], he found that states adopted more stringent emission standards than the federal Environmental Protection Agency, suggesting not only that economic competition did not motivate states to relax environmental regulations but also that there was no evidence of a “race to the bottom”. The second aspect is the type of environmental regulation competition. The “race to the bottom” is an important type of environmental regulatory competition, and the early political economy literature argues that decentralized environmental regulation is prone to the relaxation of environmental regulations to gain a competitive advantage over neighboring rivals [6]. However, Vogel is skeptical of a race to the bottom theory [7], citing the example of automobile emission standards, finding that the federal government and many state governments have adopted California’s stricter standards over the years, and calling this phenomenon the “California effect”. Fredriksson and Millimet empirically examined the strategic interaction of U.S. manufacturing abatement costs [8], confirming that this interactive behavior occurred within a two-year window of neighboring states, and found an asymmetric response pattern in which states responded to changes in abatement costs in competing states with initially stricter environmental regulations, but not in states with initially less stringent environmental regulations. Subsequently, Zhao and Bo et al. summarized and empirically analyzed environmental regulation competition using Chinese inter-provincial and urban data [9,10], respectively, in the context of Chinese decentralization. The third aspect is the impact of environmental regulation competition. With the depth of research, some scholars focus on the economic impact of environmental regulatory competition, mainly examining the role of environmental regulatory competition on economic growth, industrial structure, ecological efficiency, and employment level [11].

### 2.2. Environmental Regulation and Carbon Emissions

With global climate change, scholars have studied the influencing factors of carbon emissions, such as technological factors [12], energy structure [13], industrial structure [14], etc. In addition, the impact of economic factors on carbon emissions and the decoupling of economic development from carbon emissions have become hot topics of numerous studies to promote global low-carbon development [15,16]. Many scholars are concerned about the impact of environmental regulations on carbon emissions, and there are two main views on the impact of existing environmental regulations on carbon emissions. One view is that the increase in the intensity of environmental regulations does not effectively curb carbon emissions. Some scholars argue that environmental regulation contributes to the achievement of carbon emission reductions. Christian et al. found that carbon emissions from transport in European countries decrease as environmental legislation becomes stricter [17]. Rubayyat et al. argued that the reduction in carbon emissions in OECD countries is due to the increase in environmental taxation [18].

Nowadays, more studies consider the impact of different types of environmental regulations on carbon emissions [19]. Environmental regulation is an important part of government regulation, and scientific judgment of the type of environmental regulation tools can provide an important basis for the effect of emission reduction. Most studies classify environmental regulations as command, market, and voluntary [20]. Yang et al. analyzed the impact of environmental regulations on carbon emissions in 32 cities in Northeast China and found that economic environmental regulations have a positive relationship with carbon emissions, and command and voluntary environmental regulations can effectively suppress carbon emissions [21]. In addition, in the context of building an environmental pluralistic and shared governance system, in addition to local governments, the effect of public participation on the efficiency of environmental regulation enforcement has also been considered in many studies [22,23]. The public environmental concern effectively plays a restraining role in informal environmental regulation, and the importance of public participation in green development provides empirical evidence for a pluralistic environmental governance system. However, there are fewer studies on the impact of environmental regulation competition on carbon emissions, and there is a lack of deeper mechanistic analysis.

### 2.3. Literature Commentary

In summary, the existing literature has examined environmental regulatory competition mainly from the perspectives of firm relocation, types of strategies, and the impact of regulatory competition, but lacks consideration of central government behavioral decisions, and few of them include the central government, local governments, and neighboring local governments in a unified framework to explore the choice of environmental regulation competition strategies. In China’s national context, the central government exerts significant influence on local government behavior through performance appraisals, official promotions, and environmental inspectors. In addition, as far as carbon emissions are concerned, relevant studies mainly focus on technological innovation, cleaner production, economic structure optimization, carbon emissions trading, environmental regulation intensity, etc. Fewer studies have studied the impact of environmental regulation competition on carbon emissions from an interactive perspective. The Spatial Autoregression Model (SAR) is mainly used to estimate the environmental regulation response coefficients in the empirical analysis, ignoring the possibility that different regions may be perturbed by the same factors and that the disturbance terms are spatially autocorrelated, which shakes the assumptions of the SAR model and, thus, leads to bias in the estimation. In addition, although the literature has examined interregional environmental regulation and carbon emissions, it has been superficial and has failed to explore the deeper factors of environmental regulation competition affecting carbon emissions. Therefore, this paper will expand on the following aspects. Firstly, we set up a three-party evolutionary game model among the central government, local governments, and neighboring local governments to incorporate the central government into the unified framework of local government environmental regulation behavior and consider the influence of central government behavior on the choice of local government environmental regulation strategies. Secondly, the spatial lag term of carbon emissions and the spatial autocorrelation of disturbance terms are considered, and the Spatial Durbin Model (SDM) is established through a series of tests to analyze the environmental regulatory competition behavior of local governments under the carbon emission reduction target. Thirdly, the mechanisms of the effect of environmental regulation competition on carbon emissions are examined from three aspects: political performance assessment effect, industrial structure optimization effect, and low-carbon technology capability improvement effect, to explore the deep-seated factors of local government environmental regulation competition on carbon emissions.

## 3. Theoretical Analysis and Research Hypothesis

### 3.1. Environmental Regulation Competition Theory

**Figure 1:**
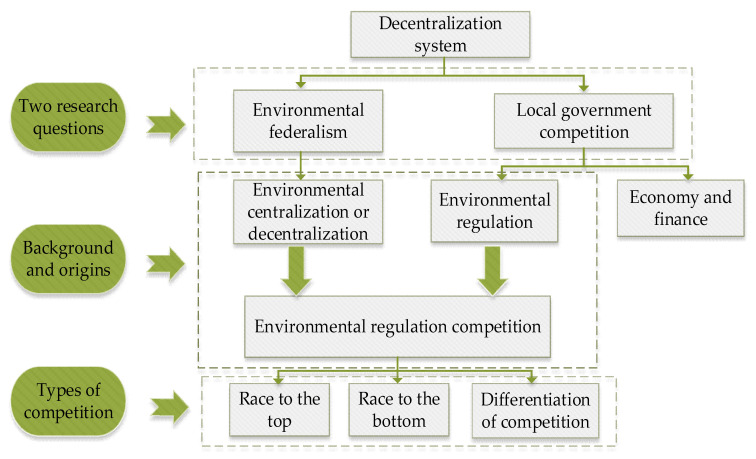
Logic diagram of environmental regulation competition theory.

Starting from the system of decentralization, this paper incorporates the theory of environmental federalism and local government competition into a unified framework to explain environmental regulatory competition, the logical framework of which is shown in Figure 1. Decentralization as a system of governance in most countries rationalizes the distribution of power in the system. There are different levels of government in the country, with higher levels of government assigning matters to lower levels of government according to certain rules, and lower authorities generally have the right to make decisions autonomously within their jurisdiction, except for major issues concerning the overall situation. It is this management structure that gives rise to two important questions for current research, one is the investigation of the degree of power distribution, and the other is the study of the competitive behavior of local governments in a decentralized system. Specifying these two questions to environmental affairs, the environment as a public good is non-competitive and non-exclusive, requiring the government to provide environmental regulation to achieve social harmonization and sustainable development. Thus, in the context of environmental federalism, regulation competition is a manifestation of local government competition in environmental regulation, and its types can be classified as “differentiation of competition”, “race to the top”, and “race to the bottom “.

As a branch of fiscal federalism, environmental federalism focuses on the issue of how much power local governments should have over environmental regulations or how to best share such control among different levels of government [24]. However, the scholarly debate on the decentralization of environmental regulations remains inconclusive. In terms of whether environmental decentralization is beneficial to the exercise of environmental regulation, one of the arguments is that information asymmetry may arise if the central government formulates local environmental policies [25,26] and that local governments are better informed within their jurisdictions than the central government and can tailor the provisions of the environmental public good to local conditions. Therefore, in addition to the national environmental affairs to the central government, local governments should be autonomous in managing the environmental affairs in their regions and can set up corresponding local standards after mastering the specific situation in their jurisdictions, which will enhance the efficiency of environmental regulation. Another view is that, compared to the information asymmetry problem, the decentralization of the environment will lead to a “race to the bottom” in environmental regulation [27]; the main reason is that local governments have unclear authority and responsibility in the provision of the public good and cross-border pollution, which ultimately leads to environmental degradation. Another important reason is that, due to the nature of the environmental public good, it is difficult for a local government alone to achieve the social optimum, so environmental matters should be managed by the central government, and local governments should play the role of implementers under the environmental standards set by the central government. Chinese fiscal decentralization, as a kind of “factual decentralization,” cannot be included in the strict sense of decentralization, and Zhang argues that the level of centralized decentralization of China’s environmental management system cannot be better measured from the fiscal perspective alone [28]; therefore, environmental federalism based on fiscal decentralization is not fully applicable in China. Starting from Oates’ theory of environmental federalism and taking into account China’s specific institutional arrangements [29], the term “Chinese-style environmental decentralization” has been derived from the study of China’s environmental management system. With the development of severe environmental problems in recent years, China’s environmental management has evolved from the initial decentralization to the current tendency of centralization, and it can be said that China’s environmental management system is both decentralized and centralized.

Local government competition refers to the competition among jurisdictions for resources to maximize local interests [30]. The core connotation of “Chinese decentralization” is “fiscal decentralization and political centralization”, which means that economic decentralization is closely integrated with the vertical political management system. On the one hand, the fiscal autonomy of local governments is based on the decentralized system, and the structure of fiscal expenditures and corresponding development policies can be chosen by local governments, while on the other hand, due to the existence of political centralization, the performance assessment focusing on economic performance will stimulate the economic competition behavior of local officials. It can be seen that the promotion system under political centralization gives local governments the incentive to compete, while fiscal decentralization gives local governments the power to compete, and some studies argue that the formation of “Chinese style yardstick competition” is inextricably linked to these two factors. With the diversification of performance evaluation, environmental regulation has also become one of the tools of local government competition. The theory of environmental regulation competition first emerged from the theory of tax competition; environmental regulation is the same as fiscal policy, which is essentially a tool for local governments to attract liquid resources. If the specific implementer of environmental regulation is the local government, then the competition of environmental regulation with the local government as a participant can be seen as a specific expression of competition in one aspect.

In summary, taking decentralization as the starting point of the study, environmental regulation competition can be seen as a product of integrating environmental federalism theory and local government competition theory [28]. Environmental regulation competition considers spatial–strategic interactions in the implementation of environmental regulation and shows that the interactions and influences between neighboring regions cannot be ignored. Neighboring regions refers to not only geographical proximity but also that local governments that are similar in the sense of economic and other development are very likely to become competitors in the “political tournament”; therefore, environmental regulatory competition is an important feature of China’s environmental policy implementation process. The types of competition concerning environmental regulation can be divided into three types. The first type of competition is “differentiation of competition”, in which one party chooses to strengthen or relax environmental regulations while the other party takes the opposite action. The second type is “race to the top”, in which one party chooses to strengthen environmental regulations and the other party also chooses to strengthen environmental regulations, which is a kind of benign competition for the provision of the environmental public good. The third category is “race to the bottom”, in which one party chooses to relax environmental regulations, and the other party also chooses to relax environmental regulations. In the second and third categories, since local governments adopt the same strategic choice in environmental regulation, they can be collectively referred to as “imitative competition”.

### 3.2. Evolutionary Game Analysis

This paper analyzes the strategic choices of the central government and two neighboring local governments in environmental regulation through an evolutionary game. Firstly, the premise of the environmental regulation game exists among the participating subjects; one is the autonomy of local governments in environmental regulation matters, and there are competing behaviors of local governments in environmental regulation under the current management system; that is, there is a game among local governments. The other is that, from the spillover effect of environmental pollutants, local governments may act incompletely or distort the implementation of the central environmental policy regulations, which may lead to inconsistent interest maximization; that is, there is a game between the central government and local governments. Secondly, regarding the choice of game method, most studies on intergovernmental environmental regulation strategy assume that the participants are perfectly rational, but because of the complexity of environmental regulatory affairs and the dynamics of the participating subjects’ continuous learning and adjusting strategies, the premise of perfect rationality is often not available, while the evolutionary game recognizes the limited rationality of the participating subjects of the game and explores the diversity of choices and preferences among subjects in the evolutionary process. Therefore, the evolutionary game model of finite rationality will be more suitable for the analysis of the behavior of the participating subjects in this paper.

#### 3.2.1. Basic Assumptions and Payoff Matrix Construction

**Assumption 1**. *The participants are central government **G**, local government **A**, and adjacent local government **B**.*

**Assumption 2**. *Each participant has two strategic choices. The strategic choice of central government **G** is active regulation or negative regulation, assuming that the probability of active regulation by the central government **G** is δ, then the probability of negative regulation is*(1−δ). *The strategic choice of local government **A** and the adjacent local government **B** is to strengthen environmental regulation or relax environmental regulation, assuming that the probability of strengthening environmental regulation by local government **A** is*α, *the probability of relaxing environmental regulation is*(1−α). *The probability of strengthening environmental regulation by the adjacent local government **B** is*β, *then the probability that environmental regulation will be relaxed is*(1−β).

When the central government adopts an active regulatory strategy, it needs to invest certain human and material resources, etc., to carry out supervision, which is the implementation cost of the central government to monitor the actions of local governments. At the same time, under the central government’s aggressive regulatory strategy, the central government will give feedback on the local government’s behavior, i.e., the local government will be rewarded for strengthening environmental regulations and punished for relaxing them. With different strategic choices by the central government, even if both local governments choose to strengthen environmental regulation, they will bring different benefits to the central government, and the benefits will be reduced if the central government does not adopt an aggressive regulatory strategy.

Similarly, there are implementation costs associated with local governments choosing to strengthen environmental regulations. Increased environmental regulation by local governments will lead to environmental improvement, while relaxed environmental regulation will lead to environmental degradation. Since this paper takes carbon dioxide emissions as the object to which environmental regulation is directed, as a pollutant with spillover effects, the actions of the two local governments influence each other. Finally, the central government’s performance appraisal of local governments is a factor that has to be taken into account because China’s performance appraisal has a great impact on local governments. The performance appraisal can be broadly divided into two categories, environmental performance appraisal and economic performance appraisal, to measure the degree of efforts made by local governments in different aspects.

The parameters of each subject in the evolutionary game model are set according to the above analysis, and their specific meanings are presented in Table 1.

To simplify the calculation, it is assumed that local governments relax environmental regulations, and the implementation cost of negative regulation by the central government is relatively zero. The benefit matrix of the three participating parties based on the above conditions is shown in Table 2.

#### 3.2.2. Stabilization Strategies for Evolutionary Games

(1) Expected benefits of central government ***G*** and replication dynamic equation.

Let $U_{G1}$ denote the expected return when central government ***G*** chooses active regulation, $U_{G2}$ denote the expected return when central government ***G*** chooses negative regulation, $\bar{U_{G}}$ denote the average expected return of central government ***G***, and $F_{G}\left(\delta \right)$ is its replication dynamic equation, which is then calculated from the payoff matrix.

(1) $$\begin{array}{cc} U_{G1} & =\alpha \beta \left(Q_{1}-C_{3}-R_{1}-R_{2}\right)+\alpha \left(1-\beta \right)\left[\left(-C_{3}\right)-R_{1}+F_{2}\right]\\ & +\left(1-\alpha \right)\beta \left[\left(-C_{3}\right)+F_{1}-R_{2}\right] \end{array}$$

(2) $$U_{G2}=\alpha \beta Q_{2}$$

(3) $$\bar{U_{G}}=\delta U_{G1}+(1-\delta )U_{G2}$$

(4) $$\begin{array}{cc} F_{G}(\delta ) & =\frac{d\delta }{dt}=\delta (U_{G1}-\bar{U_{G}})=\delta (1-\delta )(U_{G1}-U_{G2})\\ & =\delta \left(1-\delta \right)\left[\alpha \beta \left(Q_{1}+C_{3}-Q_{2}-F_{1}-F_{2}\right)+\alpha \left(F_{2}-R_{1}-C_{3}\right)+\beta \left(F_{1}-R_{2}-C_{3}\right)\right] \end{array}$$

(2) Expected benefits of local government ***A*** and replication dynamic equation.

Let $U_{A1}$ denote the expected return when local government ***A*** chooses an enhanced environmental regulation strategy, $U_{A2}$ denote the expected return when the local government chooses a relaxed environmental regulation strategy, $\bar{U_{A}}$ denote the average expected return of local government ***A***, and $F_{A}\left(\alpha \right)$ is its replicated dynamic equation, calculated from the return matrix.

(5) $$\begin{array}{cc} U_{A1} & =\beta \delta \left[\left(1-\gamma \right)\left(R_{1}-C_{1}\right)+\gamma \left(W_{1}+\theta_{2}W_{2}\right)\right]+\beta (1-\delta )\left[\left(-C_{1}\right)(1-\gamma )+\gamma (W_{1}+\theta_{2}W_{2})\right]\\ & +\left(1-\beta \right)\delta \left[\left(1-\gamma \right)\left(R_{1}-C_{1}\right)+\gamma (W_{1}-\theta_{2}P_{2})\right]+\left(1-\beta \right)\left(1-\delta \right)\left[\left(1-\gamma \right)\left(-C_{1}\right)+\gamma (W_{1}-\theta_{2}P_{2})\right] \end{array}$$

(6) $$\begin{array}{cc} U_{A2} & =\beta \delta \left[\left(1-\gamma \right)\left(-F_{1}\right)+\gamma \left(\theta_{2}W_{2}-P_{1}\right)\right]+\beta \left(1-\delta \right)\left[\gamma \left(\theta_{2}W_{2}-P_{1}\right)\right]\\ & +\left(1-\beta \right)\delta \left[\left(-\gamma \right)P_{1}-\gamma \theta_{2}P_{2}\right]+\left(1-\beta \right)\left(1-\delta \right)\left[\left(-\gamma \right)P_{1}-\gamma \theta_{2}P_{2}\right] \end{array}$$

(7) $$\bar{U_{A}}=\alpha U_{A1}+(1-\alpha )U_{A2}$$

(8) $$\begin{array}{cc} F_{A}(\alpha ) & =\frac{d\alpha }{dt}=\alpha (U_{A1}-\bar{U_{A}})=\alpha (1-\alpha )(U_{A1}-U_{A2})\\ & =\alpha \left(1-\alpha \right)\left[\beta \delta \left(1-\gamma \right)F_{1}-\left(1-\gamma \right)C_{1}+\gamma \left(W_{1}+P_{1}\right)+\delta \left(1-\gamma \right)R_{1}\right] \end{array}$$

(3) Expected benefits of local government ***B*** and replication dynamic equation.

Let $U_{B1}$ denote the expected return of local government ***B*** when it chooses an enhanced environmental regulation strategy, $U_{B2}$ denote the expected return of local government ***B*** when it chooses a relaxed environmental regulation strategy, $\bar{U_{B}}$ denote the average expected return of local government ***B***, and $F_{B}\left(\beta \right)$ is its replicated dynamic equation calculated from the return matrix.

(9) $$\begin{array}{cc} U_{B1} & =\alpha \delta \left[\left(1-\gamma \right)\left(R_{2}-C_{2}\right)+\gamma \left(W_{2}+\theta_{1}W_{1}\right)\right]+\alpha \left(1-\delta \right)\left[\left(-C_{2}\right)(1-\gamma )+\gamma (W_{2}+\theta_{1}W_{1})\right]\\ & +\left(1-\alpha \right)\delta \left[\left(1-\gamma \right)\left(R_{2}-C_{2}\right)+\gamma (W_{2}-\theta_{1}P_{1})\right]+\left(1-\alpha \right)\left(1-\delta \right)\left[\left(1-\gamma \right)\left(-C_{2}\right)+\gamma (W_{2}-\theta_{1}P_{1})\right] \end{array}$$

(10) $$\begin{array}{cc} U_{B2} & =\alpha \delta \left[\left(1-\gamma \right)\left(-F_{2}\right)+\gamma \left(\theta_{1}W_{1}-P_{2}\right)\right]+\alpha \left(1-\delta \right)\left[\gamma \left(\theta_{1}W_{1}-P_{2}\right)\right]\\ & +\left(1-\alpha \right)\delta \left[\left(-\gamma \right)P_{2}-\gamma \theta_{1}P_{1}\right]+\left(1-\alpha \right)\left(1-\delta \right)\left[\left(-\gamma \right)P_{2}-\gamma \theta_{1}P_{1}\right] \end{array}$$

(11) $$\bar{U_{B}}=\beta U_{B1}+(1-\beta )U_{B2}$$

(12) $$\begin{array}{cc} F_{B}(\beta ) & =\frac{d\beta }{dt}=\beta (U_{B1}-\bar{U_{B}})=\beta (1-\beta )(U_{B1}-U_{B2})\\ & =\beta (1-\beta )\left[\alpha \delta \left(1-\gamma \right)F_{2}-\left(1-\gamma \right)C_{2}+\gamma \left(W_{2}+P_{2}\right)+\delta \left(1-\gamma \right)R_{2}\right] \end{array}$$

Based on the replicated dynamic equations obtained, the equilibrium points of the evolutionary game are calculated as follows.

(13) $$\{\begin{array}{c} F_{G}(\delta )=\delta \left(1-\delta \right)\left[\alpha \beta \left(Q_{1}+C_{3}-Q_{2}-F_{1}-F_{2}\right)+\alpha \left(F_{2}-R_{1}-C_{3}\right)+\beta \left(F_{1}-R_{2}-C_{3}\right)\right]=0\\ F_{A}(\alpha )=\alpha \left(1-\alpha \right)\left[\beta \delta \left(1-\gamma \right)F_{1}-\left(1-\gamma \right)C_{1}+\gamma \left(W_{1}+P_{1}\right)+\delta \left(1-\gamma \right)R_{1}\right]=0\\ F_{B}(\beta )=\beta (1-\beta )\left[\alpha \delta \left(1-\gamma \right)F_{2}-\left(1-\gamma \right)C_{2}+\gamma \left(W_{2}+P_{2}\right)+\delta \left(1-\gamma \right)R_{2}\right]=0 \end{array}$$

Eight special equilibria (1, 1, 1), (0, 1, 1), (1, 1, 0), (0, 1, 0), (1, 0, 1), (0, 0, 1), (0, 0, 1), (1, 0, 0), and (0, 0, 0) can be found in Equation (13). They form the boundary of the solution domain of the evolutionary game, and the equilibrium solution ***E*** that exists represents the possible stabilization strategies adopted by local government ***A***, adjacent local government ***B*,** and central government ***G***. The local stability of the group evolution equilibrium point is solved according to the Jacobi matrix as follows.

(14) $$J=\left[\begin{array}{ccc} \frac{\partial F_{G}(\delta )}{\partial \delta } & \frac{\partial F_{G}(\delta )}{\partial \alpha } & \frac{\partial F_{G}(\delta )}{\partial \beta }\\ \frac{\partial F_{A}(\alpha )}{\partial \delta } & \frac{\partial F_{A}(\alpha )}{\partial \alpha } & \frac{\partial F_{A}(\alpha )}{\partial \beta }\\ \frac{\partial F_{B}(\beta )}{\partial \delta } & \frac{\partial F_{B}(\beta )}{\partial \alpha } & \frac{\partial F_{B}(\beta )}{\partial \beta } \end{array}\right]$$

The elements in the Jacobi matrix can be obtained based on the above analysis, which is not repeated in this paper. In this paper, we take (1, 1, 1) as an example to discuss the asymptotic stability of the system.

Assuming that the equilibrium point obtained from the game is (1, 1, 1), the eigenvalues of the matrix are solved as follows: $-\left(Q_{1}-Q_{2}-R_{1}-R_{2}-C_{3}\right)$, $-\left[\left(1-\gamma \right)\left(F_{1}-C_{1}+R_{1}\right)+\gamma \left(W_{1}+P_{1}\right)\right]$, $-\left[\left(1-\gamma \right)\left(F_{2}-C_{2}+R_{2}\right)+\gamma \left(W_{2}+P_{2}\right)\right]$. Similarly, the eigenvalues of each other equilibrium points can be obtained, as shown in Table 3.

For the equilibrium point (1, 1, 1) to be asymptotically stable, it is necessary to satisfy that all the eigenvalues obtained from the above matrix are negative. The stability of each equilibrium solution obtained from the eigenvalues is shown in Table 4.

There are six asymptotically stable points in the system (1, 1, 1), (0, 1, 1), (1, 1, 0), (0, 1, 0) (1, 0, 1), (0, 0, 1) and (0, 0, 1), and the final point of equilibrium converges depending on the initial state of the game. For example, the initial conditions required for the equilibrium point (1, 1, 1) to become asymptotically stable are $-\left(Q_{1}-Q_{2}-R_{1}-R_{2}-C_{3}\right)<0$, $-\left[\left(1-\gamma \right)\left(F_{1}-C_{1}+R_{1}\right)+\gamma \left(W_{1}+P_{1}\right)\right]<0$ and $-\left[\left(1-\gamma \right)\left(F_{2}-C_{2}+R_{2}\right)+\gamma \left(W_{2}+P_{2}\right)\right]<0$. The ideal state of equilibrium can be achieved by satisfying the above three conditions simultaneously. For the strategic choice between local governments, it is found that the performance appraisal (γ), the reward and punishment from the central government ($R_{i}$, $F_{i}$), and the effectiveness of environmental regulation for pollutants ($W_{i}$, $P_{i}$) have important effects on their strategic behavior, and the above parameters also change the costs and benefits of the central government and influence the central government’s strategic choice (i = 1, 2). Since the ideal equilibrium state is that both local governments strengthen environmental regulation under active central government regulation, the following is an example of the behavior strategy of each participant when the above conditions are not fully satisfied at the equilibrium point (1, 1, 1).

#### 3.2.3. Strategy Choice of the Game Subject

The equilibrium conditions obtained above are further analyzed to compare the strategic choices among local governments under different regulatory attitudes of the central government, and when the condition of active regulation by the central government $Q_{1}>Q_{2}+R_{1}+R_{2}+C_{3}$ is satisfied, it can be divided into the following cases.

(1) $\gamma \left(W_{1}+P_{1}\right)>\left(1-\gamma \right)\left(C_{1}-R_{1}-F_{1}\right)$, $\gamma \left(W_{2}+P_{2}\right)>\left(1-\gamma \right)\left(C_{2}-R_{2}-F_{2}\right)$, that is, when the benefits gained from environmental regulation by local government ***A*** and neighboring local government ***B*** outweigh the costs, there will be α=1, β=1. And the strategic choice of local governments will eventually evolve to strengthen environmental regulation, resulting in a “race to the top” among local governments.
(2) $\gamma \left(W_{1}+P_{1}\right)>\left(1-\gamma \right)\left(C_{1}-R_{1}-F_{1}\right)$, $\gamma \left(W_{2}+P_{2}\right)<\left(1-\gamma \right)\left(C_{2}-R_{2}-F_{2}\right)$, that is, the benefits exceed the costs when local government ***A*** environmental regulation, and the benefits are less than the costs when the adjacent local government environmental regulation, there will local government ***A*** choose to strengthen the regulation and adjacent local government ***B*** relax the regulation. Similarly, if $\gamma \left(W_{1}+P_{1}\right)<\left(1-\gamma \right)\left(C_{1}-R_{1}-F_{1}\right)$, $\gamma \left(W_{2}+P_{2}\right)>\left(1-\gamma \right)\left(C_{2}-R_{2}-F_{2}\right)$, local government ***A*** will choose to relax its environmental regulation strategy, while local government ***B*** will choose to strengthen its environmental regulation strategy, which can be regarded as “differential competition” among local governments in their choice of environmental regulation strategy.
(3) $\gamma \left(W_{1}+P_{1}\right)<\left(1-\gamma \right)\left(C_{1}-R_{1}-F_{1}\right)$, $\gamma \left(W_{2}+P_{2}\right)<\left(1-\gamma \right)\left(C_{2}-R_{2}-F_{2}\right)$, that is, the benefits of local government ***A*** and neighboring local government ***B*** when environmental regulation is less than the costs, both local government ***A*** and neighboring local government ***B*** will choose to relax environmental regulation, thus creating a “race to the bottom”.

When the central government adopts a negative regulatory attitude, the choice of environmental regulation strategy among local governments remains the same as above, except that the ideal equilibrium (1, 1, 1) in which the central government actively regulates and local governments both strengthen environmental regulation no longer exists and the system evolves to other equilibria over time.

### 3.3. Research Hypothesis

The above evolutionary game results suggest that the optimal strategy choice is made among governments by measuring the benefits and costs generated by environmental regulatory actions, and there are “race to the top”, “differentiation of competition”, and “race to the bottom”. There are three types of equilibria. Through policy analysis and literature review, this paper speculates that the overall environmental regulation competition strategy in China after the 18th National Congress is racing to the top. In the discussion of the types of environmental regulation competition, “race to the bottom” has become the view of many scholars and the focus of environmental regulation competition research because local governments are more inclined to relax environmental regulations to gain advantages in resource competition [6,31]. However, in the context of China’s environmental carrying capacity approaching the “ceiling” and the transformation of old and new dynamics, it is undesirable and unsustainable to treat environmental protection and economic growth as either/or opposites, to sacrifice the environment for economic growth or to give up development for the sake of the environment, and green development is the long-term solution. General Secretary Xi Jinping pointed out at the 2018 National Conference on Ecological Protection that “green development is an inevitable requirement for building a high-quality modern economic system and a fundamental solution to the pollution problem.” Green development aims to deal with the relationship between environmental protection and economic growth so that the two gradually form an organic cycle of positive feedback mechanism to achieve a win-win situation between economic development and environmental protection [32].

With the development of severe environmental problems in recent years, the power of local governments to make autonomous choices is firmly bound to the central government’s performance assessment. Since the 18th National Congress, China has proposed not to pollute first and then treat later and has put forward five major development concepts, all of which indicate the importance of environmental protection in China’s policy, while China has combined various tools such as environmental protection inspectors, vetoes, and binding indicators to constrain the behavior of local governments. As the performance appraisal is no longer only based on economics, local governments are less likely to relax environmental regulations to gain a more favorable position for promotion under the “baton” of performance appraisal. Moreover, in terms of pollutant management, China has achieved important results in haze and SO2 management, reflecting the increase in the intensity of environmental regulation in China. It is argued that with the change in China’s economic development model and the increase in the state’s attention to the environment, the interaction form of environmental regulation has changed from “race to the bottom” [4]. However, due to the different development situations and measures taken for environmental regulation in different places, environmental regulation competition may also vary, and regional heterogeneity exists [33]. Based on this, the following hypothesis is proposed in this paper.

**Hypothesis 1**. *Since the 18th National Congress, the competition for environmental regulations among local governments has been a “race to the top”, and there are differences in the competition for environmental regulations in different regions*.

Several scholars have found the inhibitory effect of environmental regulation enhancement on carbon emissions [34,35], and if the behavior of environmental regulation competition among local governments can affect carbon emissions, then it is necessary to analyze the mechanism of the effect of environmental regulation competition on carbon emissions. Based on literature combing [3,36,37], this paper argues that environmental regulation competition may affect carbon emissions through mechanisms such as the performance assessment effect, industrial structure optimization effect, and low-carbon technological innovation effect. Firstly, the evolutionary game analysis shows that the proportion of environmental performance assessment affects the strategy choice of local governments, which indicates that the environmental regulation behavior of local governments is constantly changing under different performance assessment incentives, which undoubtedly has an impact on carbon emissions. Secondly, industrial structure optimization is an important way to reduce carbon emissions, and finally, low-carbon technology innovation is undoubtedly a key factor in reducing carbon emissions.

Based on this, Hypothesis 2 is proposed in this paper.

**Hypothesis 2**. *Local government environmental regulation competition affects carbon emissions through the performance assessment effect, industrial structure effect, and low carbon technology innovation effect*.

## 4. Model Construction and Variable Selection

### 4.1. Econometric Model Construction

Local government competition exists between local governments in different regions, so building a spatial model is a prerequisite for empirically analyzing local government competition in environmental regulation, but the exact spatial model needs to be further tested. In this paper, we first construct three types of weight matrices to test the spatial autocorrelation of the explanatory variable carbon emissions using Moran’s *I* index: first, the geographic adjacency matrix *W*1, 1 when two regions share a common border, 0 otherwise. Second, the geographic distance matrix *W*2 is expressed as the inverse of the geographic distance obtained from the latitude and longitude coordinates of each province. Third, the geographic economic distance matrix *W*3 is calculated using the geographic distance matrix *W*2 as well as the economic distance matrix; the elements of the economic distance matrix are the inverse of the mean of the actual *GDP* per capita during the sample period. In order not to lose generality, the respective weights are taken to be 0.5. Row normalization is adopted for all three spatial matrices, and the calculation of carbon emissions is explained below. Table 5 shows the changes in Moran’s *I* index for carbon emissions in China from 2003 to 2019 under each weight matrix. It can be seen that the carbon emissions in the selected sample years all have positive spatial autocorrelation, and all of them pass the 5% significance test, indicating that carbon emissions are not only influenced by various factors in their regions but also by the surrounding areas and other factors, so their spatial correlation should not be neglected in the process of constructing the econometric model, and a spatial model with lagged terms of the explanatory variables is considered to study the interaction between carbon emissions and each province.

In addition, Greene argues that if the spatial dependence of the error term is ignored, only some efficiency loss will be caused [38], but if the relevant variables are omitted, then the estimated coefficients will be biased and non-consistent. The spatial Durbin model can alleviate the endogeneity problem caused by omitted variables to some extent and applies to most spatial data processes. Building a spatial Durbin model based on the ordinary panel model.

(15) $$\ln C_{it}=\mu +\beta_{1}Regu_{it}+\beta_{2}X_{it}+\varepsilon_{it}$$

(16) $$\ln C_{it}=\rho W_{it}\ln C_{it}+\beta_{1}Regu_{it}+\theta_{1}W_{it}Regu_{it}+\beta_{2}X_{it}+\theta_{2}W_{it}X_{it}+\mu_{i}+\gamma_{t}+\varepsilon_{it}$$

where $\ln C_{it}$ is the logarithm of carbon emissions in province *i* in year *t*, $Regu_{it}$ is the intensity of environmental regulation in province *i* in year *t*, $X_{it}$ is the remaining control variables, ρ is the spatially lagged regression coefficient, which indicates the spatial dependence of carbon emissions. $\beta_{1}$ is the local effect of environmental regulation on carbon emissions, $\theta_{1}$ is the spillover effect of environmental regulation on carbon emissions in neighboring regions, and $\beta_{2}$ is the local effect and spillover effect of the remaining control variables on carbon emissions. $\mu_{i}$, $\gamma_{t}$ is the individual effect and time effect, respectively. $\varepsilon_{it}$ is the error term, $W_{it}$ is the constructed spatial weight matrix.

After the LM test, LR test, and Wald test, it is found that SDM does not degenerate into SAR and SEM (Spatial error model), and it is found that it is more appropriate to use the fixed effect model by Hausman test, and the test results are all in Table 6. The LM test is divided into spatial error as well as spatial lag test, and both the LM test and robust LM tests pass the 5% significance level and reject the original hypothesis, which initially indicates that SDM is more suitable compared to SAR and SEM. The Hausman statistic is positive and passes the 10% significance level under the weight matrix, so the original hypothesis of random effect is rejected. Based on choosing SDM, the LR test statistics all passed the 1% significance level, and the original hypothesis was rejected. The test results concluded that the dual-fixed model was more convincing than the time-fixed or spatial-fixed model, so this paper finally chose to establish the dual-fixed spatial Durbin model.

Environmental regulation is premised on economic growth and aims to reduce pollution. According to the above theoretical analysis and with reference to the studies of Zhao and Lu [9,39], the meaning of the key coefficients $\beta_{1}$ and $\theta_{1}$ in Equation (17) is obtained and shown in Table 7. When $\beta_{1}<0$, $\theta_{1}<0$, and both are significant, the local governments show “race to the top”, it means that the local governments are competing with each other to strengthen the environmental regulations, which will have a suppressive effect on carbon emissions. When $\beta_{1}<0$, $\theta_{1}>0$, and are both significant, it means that the environmental regulations in the two regions are “differentiated competition B”, the competing government relaxes environmental regulations and the local government strengthens environmental regulations. When $\beta_{1}>0$, $\theta_{1}>0$ and are both significant, it means that the competition between local governments is a “race to the bottom”, in which the competing government relaxes environmental regulations and the local government also relaxes environmental regulations. When $\beta_{1}>0$, $\theta_{1}<0$ and are both significant, it means that there is “differentiated competition A”; it means that the competing government increases environmental regulations and the local government decreases environmental regulations. When both coefficients are insignificant, it means that there is no strategic interaction between the two places.

### 4.2. Variables and Data Selection

(1) Carbon Emissions (CO_2_)

**Figure 2:**
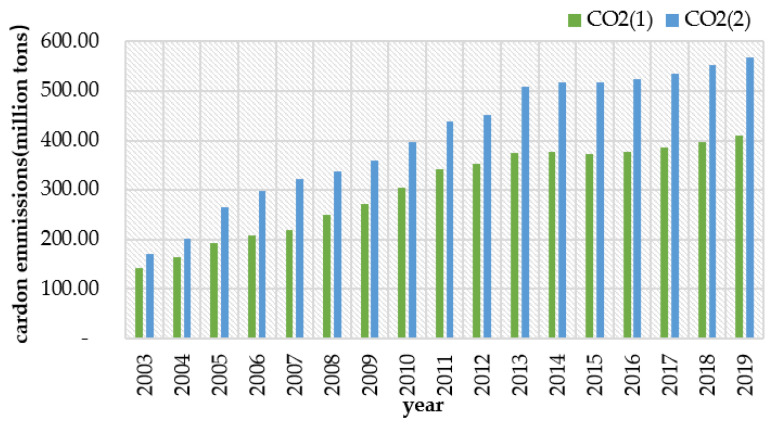
Comparison of the average carbon emission trends by region for the two measurement methods from 2003 to 2019.

Among the various greenhouse gases, CO_2_, which is the most abundant in the atmosphere, has become the focus of reduction and control. The main sources of carbon emissions are fossil fuel combustion and cement production activities. Due to the lack of direct monitoring statistics, this paper mainly refers to the carbon emission factors provided by IPCC and the research method of Du to estimate the CO_2_ produced by energy consumption and cement production [40]. The carbon emission estimation formula is:

(17) $$C_{ijt}=\sum_{i=1}^{12}E_{ijt}CEF_{j}$$

where $C_{ijt}$ denotes the total carbon emission of each province in period *t*, $E_{ijt}$ denotes the *j*th energy consumption or cement production of province *i* in period *t*, and $CEF_{j}$ denotes the carbon emission coefficient of the *j*th energy consumption or cement production. Figure 2 shows the comparison between the results of this paper (CO_2_ (2) in the figure) and the CEADs China Carbon Accounting Database (CO_2_ (1) in the figure), and the carbon emission trends are roughly the same.

(2) Environmental Regulation (*Regu*)

Existing studies on measuring environmental regulation usually use representative pollutant emissions [41], the number of environmental vocabularies [42], comprehensive indicators [43], emissions trading [44], and emission fee levy [45], etc. Emission fees as market-based environmental regulations have the characteristics of synchronous changes with other environmental policy instruments and can better measure environmental regulations [46]. This paper selects the discounted emission fee levy to measure the intensity of environmental regulation. There are three reasons for this. First, as a market-based environmental regulation, the emission fee levy is a reflection of the existing environmental policies, such as carbon emissions trading from different perspectives, and can be regarded as a comprehensive indicator [47]. Second, the target of the emission fee levy is mainly enterprises, which are the important subjects of energy consumption and thus carbon emissions, and the emission fee can reflect the low-carbon development behavior of enterprises compared with other environmental policies. The third is the continuity of the indicators. The emission charging system, which is an important part of environmental economic policy, was introduced in China in 1983, and then the environmental protection tax was introduced in 2018, the relevant policies have been implemented until now.

(3) Control variables

(1) Fiscal decentralization (*FD*). Fiscal decentralization drives local governments to compete economically, which affects population aggregation, industrial development, etc. These all affect carbon emissions. Referring to the study of Bai et al. [48], adding fiscal decentralization as a control variable and using the fiscal autonomy index to measure it. This indicator can be interpreted as the ability of local governments to finance their expenditures from their revenues and can more accurately express the division of fiscal authority between the central and local governments. (2) Energy consumption structure (*Energy*). Expressed as a ratio of coal consumption to total energy consumption. (3) Technological advances (*Tech*). Many scholars see technological progress as an important factor affecting carbon emissions [2]. Technological progress acts on high-polluting industries, which will directly reduce carbon emissions to a large extent, and the rapid growth of tertiary industries is also inseparable from technological progress, which in turn has an indirect effect on carbon emissions. In this paper, we refer to most studies to measure the level of technological development by the proportion of R&D expenditure to GDP in each province in calendar years. (4) Industrialization level (*Indu*). Expressed as a share of secondary sector output in GDP. The secondary sector mainly includes industries that are often accompanied by high pollution and energy consumption, so the level of industrialization will have an impact on carbon emissions [49]. (5) Economic Development Level (*PGDP*). At different stages of economic development, environmental pollution status and environmental protection philosophy, etc., change, which affects carbon emissions. This paper introduces the level of economic development as a control variable, measured by real gross regional product per capita [50]. (6) Population size (*pop*). Measured by calendar year-end population by province. Larger population size may bring a demographic dividend to the region with more intensive economic activities but also may consume more resources, which may have an impact on carbon emissions [51].

The data are mainly obtained from China Statistical Yearbook and China Environmental Yearbook, etc. The interpolation method is used to fill in individual missing data. The data selected in this paper span from 2003 to 2019, and all the indicators expressed in monetary units are adjusted to constant prices with 2003 as the base period. The sample selected in this paper is 30 provinces in China, and Tibet, where data are seriously missing, is not included in the sample. To prevent the interference of heteroskedasticity, logarithmic processing is carried out for some variable indicators, and the specific descriptive statistics are presented in Table 8.

## 5. Analysis of Empirical Results

### 5.1. Regression Results

The regression results of the ordinary panel and spatial Durbin models are presented in Table 9, and it can be seen that the sign of environmental regulation is negative for both the ordinary panel and SDM regressions, indicating that the increased intensity of environmental regulation has a suppressive effect on carbon emissions [52]. In contrast, the spatial model takes into account the spatial effect of environmental regulation and better reflects the competitive behavior of environmental regulation of local governments. Specifically, in the regression results using the adjacency matrix, geographic distance matrix, and geographic economic distance matrix, the coefficients of the spatial terms of environmental regulation and environmental regulation are negative and satisfy the condition of $\beta_{1}<0$ and $\theta_{1}<0$, indicating that local governments adopt a “race to the top” strategy in environmental regulation of carbon emissions, and strengthen environmental regulation in both local and neighboring places, which will have a suppressive effect on carbon emissions. If the performance appraisal system of local officials includes the assessment of environmental management, local governments have the incentive to form a “race to the top” in environmental regulation through the model of a “political tournament”.

From the end of the 20th century to the present, China’s ecological civilization has been continuously enhanced. In terms of addressing climate change, both the proposed development strategy of a “low carbon economy” and the successive implementation of “carbon peaking and carbon neutral” initiatives have demonstrated the central government’s determination to strengthen environmental regulations and reduce pollution and carbon emissions. The central government’s policy goals and preferences are applied to local governments through China’s unique decentralization system. With the establishment of the “one-vote veto system” and the strengthening of responsibility for emission reduction targets, officials’ assessment systems have incorporated binding ecological indicators, and the “China’s Policies and Actions to Address Climate Change” published in October 2021 states that provincial carbon emission control targets are established and provincial governments are assessed on their responsibility to carry out GHG emission control targets, the governance results of each jurisdiction have become the key to the competition of local officials’ performance. Numerous studies have also shown that the provincial competition for environmental regulation in China has shifted [4], and after the 18th National Congress, the ecological promotion assessment of local government officials has driven the strategic choice of local government environmental regulation from “race to the bottom” to “race to the top”.

Although the SAR model is a classical model for identifying the competitive behavior of inter-regional environmental regulation, it has some shortcomings, such as not considering the spatial correlation of the error term, the dynamic dependence of the explanatory variables, and the spatial correlation of the explanatory variables. Compared with the SAR model and spatial self-lagged (SLX) model, SDM can alleviate the endogeneity problem caused by omitted variables to a certain extent, and it takes into account the spatial correlation terms of control variables [53,54], which is applicable to most of the spatial data processes. In the study of environmental regulation competition and carbon emissions, the findings suggest that the shape of competition in environmental governance among local governments is changing from “race to the bottom”, and this change in competition strategy is closely related to the change in the direction of performance assessment [3,9,55]. As China continues to focus on carbon reduction, inter-provincial competition among local governments is optimized.

### 5.2. Robustness Tests

Considering the robustness of the obtained results, this paper uses the following methods for robustness testing: (1) Replace the explanatory variables with the carbon emission data of each province in the CEADs database to conduct the test, and the results are in Table 10. (2) Replace the environmental regulation-related indicators with the ratio of local emission fee collection to industrial value added. The results under the three spatial weight matrices are in Table 11. (3) To verify the stability of the previous findings, outliers that may be due to data calculation are excluded, and bilateral tailing at 5% quantile is performed for the explained variable (carbon emissions) as well as the core explanatory variable (environmental regulation), and the test results are presented in Table 12. (Focusing on the results of the core explanatory variables, the regression results of some control variables are not presented).

The above results show that the signs of the core explanatory variables remain stable and the results of the robustness tests are generally consistent with the previous empirical results.

### 5.3. Heterogeneity Analysis

#### 5.3.1. Heterogeneity of Subregional Samples

**Figure 3:**
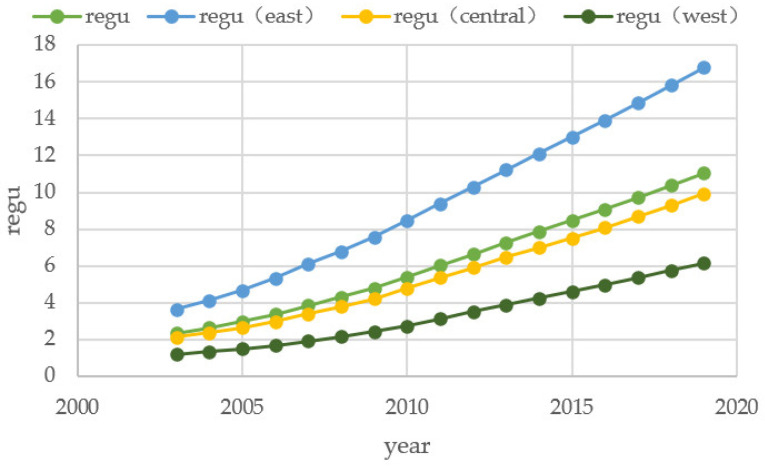
Average Environmental Regulation Intensity by East, Central, and West Regions, 2003–2019.

The above analysis shows that the competition for environmental regulations among local governments in the whole national region is a “race to the top”, and both local and neighboring local governments enhance environmental regulations to form an imitation of convergence to excellence and have a suppressive effect on the increase in carbon emissions. However, China is a vast country with different economic development and geographic conditions in each province, and regional differences cannot be ignored. From Figure 3, we find that environmental regulations in the East, Central, and West regions all show an increasing trend, which indirectly indicates the role of the “race to the top” among environmental regulations. Compared with the three regions, the intensity of environmental regulations in the East is the highest, above the national average, and the Central and Western regions are decreasing in order, so the regional differences are more obvious. In addition, the average across regions may mask differences between regions and provinces and do not preclude differences in the shape of competition in the East, Central, and West.

In this paper, the national sample is divided into three regions, Eastern, Central, and Western, for regression to determine the choice of environmental regulatory competition strategies in different regions and the differences in the impact of regional environmental regulatory competition behavior on carbon emissions in different regions. Given that there is no uniform standard for the division of China’s regions, the 30 provinces, autonomous regions, and municipalities directly under the central government studied were divided into three regions based on the division method used in the collation of statistics from the National Bureau of Statistics in 2018. The regression results are shown in Table 13.

It seems that the environmental strategies of both the Central and Western regions show a “race to the top” under the set spatial weight matrix and have a suppressive effect on carbon emissions. In the Central region, the competitive strategy is more significant under the geographic distance matrix, and the environmental regulation in the Western region shows “race to the top”, but the coefficient of local government strategy choice is not significant under the matrix except for the neighboring matrix. This is probably because the Western region is vast and the provinces and cities are far apart, so it is not accurate to consider the spatial effect of carbon emissions by using the latitude and longitude distance, and the response coefficients are different from those of the Central Provinces, indicating that the choice of environmental regulation strategies is relatively independent among the Central and Western Provinces. It is interesting to note that in the Eastern region, which is classified in the paper, the “differentiated competition” of environmental regulations appears under the adjacency matrix. This is probably because the Eastern region is divided into large areas, from Hainan in the south to northeast China, and includes municipalities such as Beijing and Shanghai, each of which performs different urban functions; the development approach and functional positioning are different, and the measures taken for environmental and economic development are different, and the competition for environmental regulation in this area appears to be differentiated. Under the geographic distance matrix and the geographic and economic distance matrix, the competition for environmental regulation in the Eastern region is a “race to the top”. In terms of the impact on carbon emission reduction, the effect of emission reduction in the Eastern region is smaller than that in the other two regions, which may be because the heavy industries and energy consumption in the Western and Central cities are higher than those in the Eastern region, and the effect of carbon emission control is soon to be seen, so the impact of environmental regulation policies on carbon emission in the Central and Western regions is more effective. As the region that plays a leading role in China’s economic development, carbon emission reduction in the Eastern region needs to be carried out gradually, and the change of development mode cannot be achieved overnight.

#### 5.3.2. Heterogeneity of the Time-Slotted Sample

The sample interval was further divided into two parts, 2003–2011 and 2012–2019. In 2012, the report of the 18th National Congress proposed to give prominence to the construction of ecological civilization, indicating that China’s environmental protection has reached a new stage. Since the 18th National Congress, China has implemented the new development concept and promoted the green transformation and low-carbon development in economic and social development. From the regression results in Table 14, the degree of inter-regional strategic interaction significantly increases between 2012 and 2019 after the implementation of stronger environmental protection policies, and the local environmental regulation also has a suppressive effect on carbon emissions in neighboring regions. The interaction of environmental regulation strategies from 2003 to 2011 is not significant, and the strengthening of environmental regulation by local and neighboring governments does not have a significant suppression effect on carbon emissions. This may be because the focus of local governments was mainly on economic development and neglected environmental protection issues, or it is most likely related to the fact that carbon emissions are not a key target constraint pollutant. With the further increase in environmental protection requirements, the strategic interaction of environmental regulation during 2012–2019 shows a “race to the top” mimicry under each spatial weight matrix, which is closer to the full-sample regression results.

### 5.4. Mechanism Analysis

To further test the mechanism of the effect of local government environmental regulation competition on carbon emissions, this paper introduces the multiplication term of performance assessment, industrial structure, and low carbon technology separately with the spatial lag of environmental regulation, respectively, to analyze, and establishes a model, as in Equation (18), $T_{it}$ denotes the mechanism variables.

(18) $$\begin{array}{cc} \ln C_{it} & =\rho W_{it}\ln C_{it}+\beta_{1}Regu_{it}+\theta_{1}W_{it}Regu_{it}+\lambda_{1}T_{it}Regu_{it}\\ & +\lambda_{2}W_{it}T_{it}Regu_{it}+\beta_{2}X_{it}+\theta_{2}W_{it}X_{it}+\mu_{i}+\gamma_{t}+\varepsilon_{it} \end{array}$$

As the “baton” and “vane” of cadre management in China, the performance appraisal plays a guiding role and incentive constraint role for local governments at all levels. For example, in 2014, the National Development and Reform Commission (NDRC), to ensure the achievement of the Twelfth Five-Year Plan carbon intensity reduction target, included the completion of carbon dioxide emission intensity reduction targets in the performance appraisal system of cadres. Under China’s centralized political and decentralized governance system, performance appraisal is usually divided into two major parts: environmental performance appraisal and economic performance appraisal [3]. On the one hand, economic performance indicators create strong incentives for local officials to develop GDP, and local governments’ efforts to promote rapid local economic development may bring about regulatory failure and environmental degradation, while on the other hand, environmental performance indicators force officials to deal with the relationship between environmental protection and economic development to obtain a promotion, strengthening the possibility of healthy environmental pollution control competition among local governments. Therefore, the requirements of these two types of performance appraisals determine the direction of the development of local government strategy interaction, and the optimization of the content of performance appraisals will enhance the motivation of local governments to strengthen environmental regulation, which will have a great boosting effect on carbon emission control.

To test the effect of performance appraisal, the performance appraisal was divided into two aspects: economic performance appraisal and environmental performance appraisal, by referring to Zhang [3]. Economic performance (*SYSg*) is expressed as the difference between the provincial economic growth rate and the national economic growth rate, and the larger its value indicates the better economic performance of the province. Environmental performance (*SYSe*) is expressed as the opposite of the difference between the provincial carbon emission of CNY 10,000 GDP and the national carbon emission of CNY 10,000 GDP, and the larger its value indicates the better environmental performance of the province. The regression results of the effect of political performance assessment are shown in Table 15. The coefficient of the multiplication term between environmental regulation and economic performance assessment is positive and has relatively small values, and the improvement of economic performance assessment has a relatively weak effect on carbon emissions, indicating that economic development and carbon emission reduction are not opposites, and a win-win situation exists for both high-quality economic development and carbon emission reduction. The multiplication term coefficient of environmental regulation and environmental performance assessment is significantly negative, indicating that the enhancement of local environmental performance has a suppressive effect on carbon emissions, environmental regulations reduce carbon emissions in neighboring areas through environmental performance assessment. In summary, the environmental performance assessment strengthens the “race to the top” of local governments, and the performance assessment effect caused by the competition for environmental regulations is more reflected in the environmental performance.

The industrial structure optimization effect (*Stru*) is expressed by the proportion of the output value of the tertiary industry to the output value of the secondary industry, and the low carbon technology capability improvement effect (*Lcte*) is measured by the growth rate of low carbon technology patent applications, and the regression results are shown in Table 16. For the industrial structure optimization effect, under the geographic distance matrix and the geographic economic distance matrix, the multiplication term coefficients of environmental regulation and industrial structure and the spatial multiplication term are significant; the negative coefficients indicate that environmental regulation competition promotes carbon emission reduction through the industrial structure optimization effect. The energy consumed by industries, etc., is the main source of carbon emissions, so the implementation of strict environmental regulations can force industrial restructuring. On the one hand, stringent environmental regulations make high energy-consuming and high-polluting industries bear high “environmental compliance costs”, and to avoid this cost, pollution-intensive industries will move to areas with less stringent environmental regulations. On the other hand, clean industries will not be affected by such a situation, so the severe environmental regulations will effectively inhibit the expansion of pollution-intensive industries and promote the development of clean industries, such as service industries, which will lead to the development of advanced industrial structure, and the carbon emissions will be reduced.

As for the effect of low-carbon technology capability improvement, the multiplication term coefficient and spatial multiplication term coefficient of environmental regulation and low-carbon technology innovation are negative and significant. This indicates that environmental regulation competition can lead to the upgrading of production technology through low-carbon technology capability improvement, which, in turn, has a dampening effect on carbon emissions. The Porter hypothesis suggests that appropriate environmental regulations can stimulate low-carbon technology innovation and promote the upgrading of environmental technology to effectively reduce carbon emissions. At the same time, there is a “compensating effect” between environmental regulation and low-carbon technology innovation, in which technological upgrading will enhance the competitive advantage of enterprises in the market, which not only compensates for the cost of environmental compliance but also promotes the development of enterprise technology innovation, thus realizing the synergistic development of production efficiency and environmental quality improvement. In summary, environmental regulation competition reduces carbon emissions by optimizing the industrial structure and improving low-carbon technology capabilities.

## 6. Conclusions

In this paper, an evolutionary game model of the central government and two neighboring local governments is constructed to discuss the behavioral strategies of the participating agents. The spatial Durbin model is then used to test the impact of local government environmental regulation competition on carbon emissions. The main conclusions are as follows. (1) There are three types of equilibria in the evolutionary game, namely, “race to the top”, “race to the bottom”, and “differentiated competition”; the ideal equilibrium of the game is related to the benefits and costs of the behavioral strategies of each participant, among which the performance appraisal has an important influence on the environmental regulation behavior of local governments. (2) The empirical results show that the competition among local governments for environmental regulations is a “race to the top “; that is, the local government strengthens environmental regulations, and the neighboring regions also strengthen environmental regulations, and the competition results have a suppressive effect on carbon emissions. (3) Heterogeneity analysis shows that the environmental competition strategies of both Central and Western regions are “race to the top “, and the Central region has the most obvious effect on emission reduction under the set weight matrix. Since the 18th National Congress, the interaction of environmental regulation strategies between regions has increased significantly, and environmental regulation in the region has a suppressive effect on carbon emissions in neighboring regions. Environmental regulation competition affects carbon emissions through the performance assessment effect, industrial structure optimization effect, and low-carbon technology capability improvement effect.

The following recommendations are made in conjunction with the above findings. (1) Follow the local governments’ interest function and balance the interests of all parties to achieve the emissions reduction target. The behavioral choices of local governments are related to their benefits and costs, and carbon emissions have significant spillover, so collaborative regional governance is crucial. First, the central government should build a reasonable internal incentive mechanism, such as ecological compensation transfer payments, to coordinate the interests of all parties and enhance the incentive of local governments to enhance environmental regulation. Second, promoting cross-regional joint prevention and control governance requires multiple efforts, and local governments should not be the only subject to perform environmental regulation duties; enterprises and the public in the region all have a social responsibility for greenhouse gas management and should cultivate the concept of win-win cooperation and work together to strengthen the convergent competition among local governments. (2) Appropriately increase the proportion of environmental performance assessment and optimize the performance assessment system. The central government has a leading role in the behavior strategy of local governments, and a proper performance appraisal system will motivate local governments to achieve the goal of environmental protection. First, the central government needs to improve the assessment standards for local government officials and increase the proportion of environmental protection in the local performance appraisal system in the face of the performance logic followed by local officials, and appropriately reduce the assessment indexes related to GDP growth, etc., so that the performance appraisal can correctly lead the behavioral choices of local governments. Second, expenditure responsibilities need to be reasonably divided to enhance the financial transparency of local governments, establish a more reasonable fiscal balance and regional policy system, help the implementation of environmental regulations, strengthen environmental protection inspectors, deliver a clear message that local environmental performance should not be neglected while paying attention to economic performance, and promote a win-win situation for both economic growth and carbon emission reduction. (3) Consider regional development differences and find the right carbon emission reduction path. First, based on economic development, low-carbon development targets are set in batches and targeted, and the decomposition mechanism of each region is refined. Given the distinctive characteristics of China’s regional development and the realistic disparities in the development levels of different regions, regional differences need to be fully considered, and environmental regulation strategies should be selected according to local conditions to promote ecological and economic co-development. Second, according to the actual local adjustment of industrial structure, the development of low-carbon industries, guidance to industry to adjust the input mix, design good, phased, progressive industrial optimization goals, promote the synchronization of management systems and policy reform, to facilitate the registration of low-carbon enterprises. Third, to build a mechanism for cooperation between industry, academia, and research in line with regional development, guide large enterprises and state-owned enterprises to play a leading role in innovation, increase investment in clean energy science and technology, improve the innovation system in the field of low-carbon technologies, and support enterprises in technological transformation and technological research through interest subsidies, tax breaks, financial support, and other measures.

## Figures and Tables

**Table 1:** Parameters of each subject of the three-party evolutionary game and their meanings.

Parameters	Meaning
$C_{3}$	The cost of monitoring local government implementation when central government ***G*** adopts an active regulatory strategy.
$R_{1}$	When the central government actively regulates, local government ***A*** is rewarded for strengthening environmental regulations.
$R_{2}$	When the central government actively regulates, local government ***B*** is rewarded for strengthening environmental regulations.
$F_{1}$	When the central government actively regulates, local government ***A*** is punished by the central government for relaxing environmental regulations.
$F_{2}$	When the central government actively regulates, local government ***B*** is punished by the central government for relaxing environmental regulations.
$Q_{1}$	Benefits to the central government from increased environmental regulation by the two local governments when the central government actively regulates.
$Q_{2}$	Benefits to the central government from increased environmental regulation by the two local governments when the central government is negatively regulating.
$C_{1}$	Local government ***A*** enhances the enforcement costs of environmental regulations.
$W_{1}$	The extent of environmental improvement resulting from enhanced environmental regulation by local government ***A***.
$P_{1}$	The extent of environmental degradation brought about by the diminish of environmental regulations by local government ***A***.
$\theta_{1}$	Impact of improved or deteriorating environment of local government ***A*** on adjacent local government ***B***.
$C_{2}$	Local government ***B*** enhances the enforcement costs of environmental regulations.
$W_{2}$	The extent of environmental improvement resulting from enhanced environmental regulation by local government ***B***.
$P_{2}$	The extent of environmental degradation brought about by the diminished environmental regulations by the adjacent local government ***B***.
$\theta_{2}$	Impact of improved or deteriorating environment of local government ***B*** on adjacent local government ***A***.
γ	The proportion of environmental performance assessment in the central government’s performance appraisal of local governments.
1−γ	The proportion of economic performance assessment in the central government’s performance appraisal of local governments.

**Table 2:** Strategy combinations and payoff matrix of the participants of the three-party game.

Strategy Combination	The Central Government *G*	Local Government *A*	Adjacent Local Government *B*
(Active, Enhanced, Enhanced)	$Q_{1}-C_{3}-R_{1}-R_{2}$	$\left(1-\gamma \right)\left(R_{1}-C_{1}\right)+\gamma \left(W_{1}+\theta_{2}W_{2}\right)$	$\left(1-\gamma \right)\left(R_{2}-C_{2}\right)+\gamma \left(W_{2}+\theta_{1}W_{1}\right)$
(Negative, Enhanced, Enhanced)	$Q_{2}$	$-C_{1}(1-\gamma )+\gamma (W_{1}+\theta_{2}W_{2})$	$-C_{2}(1-\gamma )+\gamma (W_{2}+\theta_{1}W_{1})$
(Active, Enhanced, Diminish)	$-C_{3}-R_{1}+F_{2}$	$\left(1-\gamma \right)\left(R_{1}-C_{1}\right)+\gamma (W_{1}-\theta_{2}P_{2})$	$\left(1-\gamma \right)\left(-F_{2}\right)+\gamma \left(\theta_{1}W_{1}-P_{2}\right)$
(Negative, Enhanced, Diminish)	0	$\left(1-\gamma \right)\left(-C_{1}\right)+\gamma (W_{1}-\theta_{2}P_{2})$	$\gamma \left(\theta_{1}W_{1}-P_{2}\right)$
(Active, Diminished, Enhanced)	$-C_{3}+F_{1}-R_{2}$	$\left(1-\gamma \right)\left(-F_{1}\right)+\gamma \left(\theta_{2}W_{2}-P_{1}\right)$	$\left(1-\gamma \right)\left(R_{2}-C_{2}\right)+\gamma (W_{2}-\theta_{1}P_{1})$
(Negative, Diminished, Enhanced)	0	$\gamma \left(\theta_{2}W_{2}-P_{1}\right)$	$\left(1-\gamma \right)\left(-C_{2}\right)+\gamma (W_{2}-\theta_{1}P_{1})$
(Active, Diminished, Diminished)	0	$-\gamma P_{1}-\gamma \theta_{2}P_{2}$	$-\gamma P_{2}-\gamma \theta_{1}P_{1}$
(Negative, Diminished, Diminished)	0	$-\gamma P_{1}-\gamma \theta_{2}P_{2}$	$-\gamma P_{2}-\gamma \theta_{1}P_{1}$

**Table 3:** Eigenvalues of the equilibrium solution.

Balanced Solution	Matrix Eigenvalues
(1, 1, 1)	$-\left(Q_{1}-Q_{2}-R_{1}-R_{2}-C_{3}\right)$, $-\left[\left(1-\gamma \right)\left(F_{1}-C_{1}+R_{1}\right)+\gamma \left(W_{1}+P_{1}\right)\right]$, $-\left[\left(1-\gamma \right)\left(F_{2}-C_{2}+R_{2}\right)+\gamma \left(W_{2}+P_{2}\right)\right]$
(0, 1, 1)	$Q_{1}-Q_{2}-R_{1}-R_{2}-C_{3}$, $\left(1-\gamma \right)C_{1}-\gamma \left(W_{1}+P_{1}\right)$, $\left(1-\gamma \right)C_{2}-\gamma \left(W_{2}+P_{2}\right)$
(1, 1, 0)	$-\left(F_{2}-R_{1}-C_{3}\right)$, $-\left[\left(1-\gamma \right)\left(R_{1}-C_{1}\right)+\gamma \left(W_{1}+P_{1}\right)\right]$, $\left(1-\gamma \right)\left(F_{2}-C_{2}+R_{2}\right)+\gamma \left(W_{2}+P_{2}\right)$
(0, 1, 0)	$F_{2}-R_{1}-C_{3}$, $\left(1-\gamma \right)C_{1}-\gamma \left(W_{1}+P_{1}\right)$, $-\left(1-\gamma \right)C_{2}+\gamma \left(W_{2}+P_{2}\right)$
(1, 0, 1)	$-\left(F_{1}-R_{2}-C_{3}\right)$, $\left(1-\gamma \right)\left(F_{1}-C_{1}+R_{1}\right)+\gamma \left(W_{1}+P_{1}\right)$, $-\left[\left(1-\gamma \right)\left(R_{2}-C_{2}\right)+\gamma \left(W_{2}+P_{2}\right)\right]$
(0, 0, 1)	$F_{1}-R_{2}-C_{3}$, $-\left(1-\gamma \right)C_{1}+\gamma \left(W_{1}+P_{1}\right)$, $\left(1-\gamma \right)C_{2}-\gamma \left(W_{2}+P_{2}\right)$
(1, 0, 0)	0, $\left(1-\gamma \right)\left(R_{1}-C_{1}\right)+\gamma \left(W_{1}+P_{1}\right)$, $\left(1-\gamma \right)\left(R_{2}-C_{2}\right)+\gamma \left(W_{2}+P_{2}\right)$
(0, 0, 0)	0, $-\left(1-\gamma \right)C_{1}+\gamma \left(W_{1}+P_{1}\right)$, $-\left(1-\gamma \right)C_{2}+\gamma \left(W_{2}+P_{2}\right)$

**Table 4:** Stability of the equilibrium solution.

No.	Balanced Solution	Eigenvalue Symbols	Stability
1	(1, 1, 1)	All negative values are possible	Asymptotic stability point
2	(0, 1, 1)	All negative values are possible	Asymptotic stability point
3	(1, 1, 0)	All negative values are possible	Asymptotic stability point
4	(0, 1, 0)	All negative values are possible	Asymptotic stability point
5	(1, 0, 1)	All negative values are possible	Asymptotic stability point
6	(0, 0, 1)	All negative values are possible	Asymptotic stability point
7	(1, 0, 0)	Not all negative values	Instability point
8	(0, 0, 0)	Not all negative values	Instability point

**Table 5:** Moran’s *I* index of carbon emissions under each spatial weight matrix.

Weighting Matrix	Year	Variable	I	E(I)	sd(I)	z	*p*-Value
*W*1	y2003	CO_2_	0.260	−0.034	0.109	2.709	0.003
	y2010	CO_2_	0.294	−0.034	0.109	3.013	0.001
	y2015	CO_2_	0.250	−0.034	0.108	2.638	0.004
	y2019	CO_2_	0.201	−0.034	0.108	2.173	0.015
*W*2	y2003	CO_2_	0.072	−0.034	0.038	2.784	0.003
	y2010	CO_2_	0.051	−0.034	0.038	2.235	0.013
	y2015	CO_2_	0.043	−0.034	0.038	2.039	0.021
	y2019	CO_2_	0.033	−0.034	0.038	1.758	0.039
*W*3	y2003	CO_2_	0.050	−0.034	0.060	1.403	0.080
	y2010	CO_2_	0.063	−0.034	0.060	1.626	0.052
	y2015	CO_2_	0.062	−0.034	0.060	1.616	0.053
	y2019	CO_2_	0.065	−0.034	0.060	1.663	0.048

Note: Due to space limitations, only the Moran test results for 2003, 2010, 2015, and 2019 were selected.

**Table 6:** Correlation test results under each spatial weight matrix.

LM Test	*W*1		*W*2		*W*3	
	**Statistic**	***p*-Value**	**Statistic**	***p*-Value**	**Statistic**	***p*-Value**
Spatial error:						
Moran’s I	23.250	0.000	42.876	0.000	36.757	0.000
Lagrange multiplier	507.999	0.000	1489.173	0.000	1140.620	0.000
Robust Lagrange multiplier	264.540	0.000	1177.448	0.000	887.862	0.000
Spatial lag:						
Lagrange multiplier	250.685	0.000	318.304	0.000	257.913	0.000
Robust Lagrange multiplier	7.226	0.007	6.579	0.010	5.156	0.023
**Hausman Test**	***W*1**		***W*2**		***W*3**	
Assumption:						
Ho: difference in coeffs not systematic	19.99	0.1725	22.44	0.0967	21.20	0.1304
**LR Test**	***W*1**		***W*2**		***W*3**	
Assumption:						
SAR nested in SDM	49.08	0.000	49.60	0.0000	48.65	0.0000
SEM nested in SDM	49.34	0.000	48.95	0.0000	48.93	0.0000
Assumption:						
SDM (time) nested in SDM	783.42	0.0000	831.03	0.0000	887.17	0.0000
SDM (ind) nested in SDM	95.46	0.0000	60.37	0.0000	55.36	0.0000
**Wald Test**	***W*1**		***W*2**		***W*3**	
	13.300	0.065	14.63	0.041	17.18	0.0163

**Table 7:** Symbols and meanings of environmental regulation coefficients.

The Symbols of $\beta_{1}$	The Symbols of $\theta_{1}$	Meanings
+	+	Race to the Bottom
+	–	Differentiated Competition A
+	/	No strategy interaction
–	+	Differentiated Competition B
–	–	Race to the Top
–	/	No strategy interaction
/	+	No strategy interaction
/	–	No strategy interaction
/	/	No strategy interaction

Note: + indicates a positive effect, – indicates a negative effect, and / indicates not significant.

**Table 8:** Descriptive statistics of the variables (N = 510).

Variable Type	Variable Name	Average Value	Standard Deviation	Minimum Value	Maximum Value
Explained variables	Carbon Emissions (*lnC*)	5.733066	0.8038663	2.87547	7.44787
Core explanatory variables	Environmental Regulation (*Regu*)	6.241596	6.190122	0.0866	40.02529
Other Variables	Fiscal Decentralization (*FD*)	0.5087291	0.1906643	0.1482647	0.9508641
	Energy Structure (*lnEnergy*)	4.092285	0.4681441	0.0238004	4.583137
	Technological Advances (*Tech*)	1.835614	1.240507	0.3474509	8.864757
	Industrial Ratio (*Indu*)	48.65343	9.253797	21.35912	69.52912
	Economic Development Level (*lnPGDP*)	9.942368	0.6941139	8.006894	11.54742
	Population Size (*lnPop*)	8.173519	0.7489935	6.280396	9.351927
	Economic Performance Assessment (*SYSg*)	6.57735	13.06446	−105.2294	54.38801
	Environmental Performance Assessment (*SYSe*)	−2.344079	15.26785	−79.5948	38.13917
	Industry Structure (*Stru*)	3.585665	1.180086	−0.2862951	6.136548
	Low Carbon Technology Innovation (*Lcte*)	0.3569702	0.4981338	−0.68	5.326087

**Table 9:** Regression results of the effect of local government environmental regulation competition on carbon emissions (N = 510).

	(1)	(2)	(3)	(4)
Explanatory Variables	Panel Model	Geographic Adjacency *W*1	Geographical Distance *W*2	Geographical Economy *W*3
*Regu*	−0.009 ***	−0.012 ***	−0.015 ***	−0.011 ***
	(0.003)	(0.003)	(0.003)	(0.003)
*FD*	0.112	0.009	0.189	0.142
	(0.160)	(0.222)	(0.221)	(0.218)
*lnEnergy*	0.081 **	0.103 ***	0.093 ***	0.063 **
	(0.039)	(0.032)	(0.032)	(0.031)
*Tech*	0.038 ***	0.030	0.060 ***	0.047 ***
	(0.010)	(0.021)	(0.020)	(0.021)
*Indu*	0.005 **	0.007 *	0.005	−0.002
	(0.006)	(0.004)	(0.004)	(0.634)
*lnPGDP*	0.209	0.434 **	−0.373 *	0.402 **
	(0.250)	(0.185)	(0.199)	(0.164)
*lnPop*	0.484 ***	0.952 ***	0.997 ***	0.960 ***
	(0.102)	(0.270)	(0.232)	(0.241)
*W* × *Regu*		−0.015 **	−0.121 ***	−0.098 ***
		(0.007)	(0.025)	(0.024)
*W* × *FD*		0.717^*^	2.127	1.097 ***
		(0.431)	(1.427)	(1.051)
Control variables	yes	yes	yes	yes
Time fixed effect	yes	yes	yes	yes
Space fixed effect	yes	yes	yes	yes
*rho*		0.069	−0.182	0.061 *
		(0.066)	(0.185)	(0.146)
*sigma2_e*		0.017 ***	0.017 ***	0.017 ***
		(0.001)	(0.001)	(0.001)
*R-squared*	0.850	0.129	0.0144	0.0059
*Log-likelihood*		310.389	310.364	310.019
*Number of ID*	30	30	30	30

Note: Values in parentheses are standard errors, *** *p* < 0.01, ** *p* < 0.05, * *p* < 0.1. Results for explanatory variables are not fully presented.

**Table 10:** Robustness test results under each spatial weight matrix of replacement carbon emission index.

	(1)	(2)	(3)
Variables	*W*1	*W*2	*W*3
*Regu*	−0.026 ***	−0.026 ***	−0.024 ***
	(0.004)	(0.004)	(0.004)
*FD*	−0.489 *	−0.146	−0.088
	(0.269)	(0.270)	(0.265)
*W* × *Regu*	−0.009	−0.076 **	−0.086 ***
	(0.008)	(0.030)	(0.030)
*W* × *FD*	0.651	−1.299	−0.609
	(0.523)	(1.751)	(1.286)
*rho*	0.111	−0.156	0.126
	(0.072)	(0.183)	(0.138)
Control variables	yes	yes	yes
Time fixed effect	yes	yes	yes
Space fixed effect	yes	yes	yes
*sigma2_e*	0.026 ***	0.026 ***	0.026 ***
	(0.001)	(0.002)	(0.002)
*R-squared*	0.111	0.086	0.091
*Log-likelihood*	210.946	208.005	211.391
*Number of ID*	30	30	30

Note: Values in parentheses are standard errors, *** *p* < 0.01, ** *p* < 0.05, * *p* < 0.1.

**Table 11:** Robustness test results under each spatial weight matrix of replacement environmental regulation indicators.

	(1)	(2)	(3)
Variables	*W*1	*W*2	*W*3
*rRegu*	−0.214 *	−0.250 *	−0.033
	(0.123)	(0.132)	(0.130)
*FD*	0.079	0.152	0.182
	(0.198)	(0.202)	(0.205)
*W* × *rRegu*	−0.715 *	−3.306 ***	−0.382
	(0.304)	(1.010)	(0.288)
*W* × *FD*	1.204 ***	4.138 ***	1.980 ***
	(0.375)	(1.297)	(0.611)
*rho*	0.022	−0.164	0.055
	(0.066)	(0.182)	(0.076)
Control variables	yes	yes	yes
Time fixed effect	yes	yes	yes
Space fixed effect	yes	yes	yes
*sigma2_e*	0.015 ***	0.016 ***	0.016 ***
	(0.001)	(0.001)	(0.001)
*R-squared*	0.084	0.055	0.296
*Log-likelihood*	341.561	328.210	327.781
*Number of ID*	30	30	30

Note: Values in parentheses are standard errors, *** *p* < 0.01, * *p* < 0.1.

**Table 12:** Robustness test results under each spatial weight matrix after the tailing process.

	(1)	(2)	(3)
Variables	*W*1	*W*2	*W*3
*Regu*	−0.27 ***	−0.034 ***	−0.030
	(0.004)	(0.005)	(0.005)
*FD*	0.067	0.258	0.251
	(0.211)	(0.212)	(0.215)
*W* × *Regu*	−0.049 ***	−0.246 ***	−0.144
	(0.010)	(0.040)	(0.031)
*W* × *FD*	1.176 ***	2.219	2.015 ***
	(0.401)	(1.365)	(1.018)
*rho*	−0.031	−0.249	−0.012
	(0.068)	(0.188)	(0.150)
Control variables	yes	yes	yes
Time fixed effect	yes	yes	yes
Space fixed effect	yes	yes	yes
*sigma2_e*	−0.016 ***	0.016 ***	0.016 ***
	(0.001)	(0.001)	(0.001)
*R-squared*	0.051	0.056	0.043
*Log-likelihood*	332.443	328.029	324.550
*Number of ID*	30	30	30

Note: Values in parentheses are standard errors, *** *p* < 0.01.

**Table 13:** Regression results of the impact of environmental regulation competition on carbon emissions by region.

	(1)	(2)	(3)	(4)	(5)	(6)	(7)	(8)	(9)
	East			Central			West		
Variables	*W*1	*W*2	*W*3	*W*1	*W*2	*W*3	*W*1	*W*2	*W*3
*Regu*	−0.005 **	−0.034 ***	−0.034 ***	−0.066 ***	−0.093 ***	−0.096 ***	−0.082 ***	−0.028	−0.028
	(0.003)	(0.011)	(0.011)	(0.006)	(0.012)	(0.011)	(0.019)	(0.025)	(0.025)
*FD*	0.446 **	0.259 *	0.259 *	−0.294	−0.146 ***	−0.124	0.892	1.389 **	1.386 **
	(0.199)	(0.139)	(0.139)	(0.180)	(0.175)	(0.170)	(0.481)	(0.568)	(0.568)
*W* × *Regu*	0.013 ***	−0.142 ***	−0.142 ***	−0.015 *	−0.193 ***	−0.192 ***	−0.236 ***	−0.332 **	−0.332 **
	(0.005)	(0.055)	(0.055)	(0.009)	(0.048)	(0.044)	(0.050)	(0.145)	(0.145)
*W* × *FD*	0.457	0.068	0.068	1.330 ***	2.543 ***	2.640 ***	−0.466	3.167	3.153
	(0.340)	(0.573)	(0.573)	(0.274)	(0.579)	(0.576)	(0.979)	(2.803)	(2.803)
Control variables	yes	yes	yes	yes	yes	yes	yes	yes	yes
Time fixed effect	yes	yes	yes	yes	yes	yes	yes	yes	yes
Space fixed effect	yes	yes	yes	yes	yes	yes	yes	yes	yes
*rho*	0.060 **	−0.306 *	−0.306 *	−0.256 ***	−0.689 ***	−0.740 ***	−0.260 **	−0.462 **	−0.462 **
	(0.079)	(0.177)	(0.177)	(0.084)	(0.165)	(0.164)	(0.102)	(0.209)	(0.209)
*sigma2_e*	0.006 ***	0.004 ***	0.004 ***	0.002 ***	0.002 ***	0.001 ***	0.0134 ***	0.015 ***	0.015 ***
	(0.001)	(0.001)	(0.001)	(0.000)	(0.000)	(0.000)	(0.001)	(0.002)	(0.002)
*R-squared*	0.321	0.041	0.041	0.115	0.034	0.028	0.635	0.646	0.645
*Log-likelihood*	210.210	239.629	239.624	−40.784	240.373	242.522	137.958	124.311	124.404
*Number of ID*	11	11	11	8	8	8	11	11	11

Note: The values in parentheses are standard errors, *** *p* < 0.01, ** *p* < 0.05, * *p* < 0.1.

**Table 14:** Regression results of the impact of environmental regulation competition on carbon emissions by period.

	(1)	(2)	(3)	(4)	(5)	(6)
	2003–2011			2012–2019		
Variables	*W*1	*W*2	*W*3	*W*1	*W*2	*W*3
*Regu*	0.018 **	0.012	0.004	−0.986 **	−1.501 ***	−1.619 ***
	(0.008)	(0.008)	(0.009)	(0385)	(0.333)	(0.347)
*FD*	0.101	0.316	0.184	0.378	0.481 **	0.333
	(0.241)	(0.250)	(0.255)	(0.250)	(0.242)	(0.252)
*W* × *Regu*	0.028 **	0.135 *	0.127 *	−2.260 ***	−5.961 **	−3.472 *
	(0.015)	(0.070)	(0.070)	(0.768)	(2.391)	(2.008)
*W* × *FD*	1.292 **	1.625	3.377 **	0.199	2.326 **	0.016
	(0.514)	(2.004)	(1.650)	(0.441)	(1.499)	(1.497)
Control variables	yes	yes	yes	yes	yes	yes
Time fixed effect	yes	yes	yes	yes	yes	yes
Space fixed effect	yes	yes	yes	yes	yes	yes
*rho*	−0.238 ***	−0.243	−0.096	−0.005	−0.522 *	−0.201
	(0.092)	(0.251)	(0.208)	(0.094)	(0.270)	(0.222)
*sigma2_e*	0.010 ***	0.011 ***	0.011 ***	0.006 ***	0.006 ***	0.006 ***
	(0.001)	(0.001)	(0.001)	(0.001)	(0.001)	(0.001)
*R-squared*	0.123	0.157	0.028	0.394	0.333	0.324
*Log-likelihood*	240.529	225.359	222.359	272.234	276.909	270.687
*Number of ID*	30	30	30	30	30	30

Note: Values in parentheses are standard errors, *** *p* < 0.01, ** *p* < 0.05, * *p* < 0.1.

**Table 15:** Performance appraisal effect test.

	(1)	(2)	(3)
	*W*1	*W*2	*W*3
*Regu*	−0.005 ***	−0.006 ***	−0.004 **
	(0.002)	(0.002)	(0.002)
*Regu × SYSg*	0.001 **	0.000	0.001 ***
	(0.000)	(0.000)	(0.000)
*Regu × SYSe*	−0.028 ***	−0.030 ***	−0.028 ***
	(0.001)	(0.001)	(0.001)
*W × Regu*	−0.005 *	−0.047 ***	−0.025 **
	(0.003)	(0.013)	(0.011)
*W × Regu × SYSg*	0.000	0.006 **	0.004
	(0.001)	(0.003)	(0.002)
*W × Regu × SYSe*	−0.005 **	−0.016 **	−0.020 ***
	(0.002)	(0.008)	(0.008)
Control variables	yes	yes	yes
Time fixed effect	yes	yes	yes
Space fixed effect	yes	yes	yes
*Rho*	−0.191 ***	−0.007	−0.278 *
	(0.069)	(0.169)	(0.151)
*sigma2_e*	0.006 ***	0.004 ***	0.006 ***
	(0.001)	(0.001)	(0.001)
*R-squared*	0.014	0.029	0.058
*Log-likelihood*	595.541	666.375	592.828
*Number of ID*	30	30	30

Note: Values in parentheses are standard errors, *** *p* < 0.01, ** *p* < 0.05, * *p* < 0.1.

**Table 16:** Test of industrial structure optimization effect and low carbon technology capability improvement effect.

	(1)	(2)	(3)	(4)	(5)	(6)
	*W*1	*W*2	*W*3	*W*1	*W*2	*W*3
*Regu*	−0.012 ***	−0.016 ***	−0.012 ***	−0.015 ***	−0.016 ***	−0.014 ***
	(0.003)	(0.003)	(0.003)	(0.004)	(0.003)	(0.003)
*Regu × Stru*	−0.235 ***	−0.392 ***	−0.320 ***			
	(0.058)	(0.064)	(0.058)			
*Regu × Lcte*				−0.009 *	−0.008 *	−0.007
				(0.004)	(0.005)	(0.005)
*W × Regu*	−0.015 **	−0.141 ***	−0.133 ***	−0.014 *	−0.066 ***	−0.058 ***
	(0.007)	(0.025)	(0.024)	(0.008)	(0.021)	(0.020)
*W × Regu × Stru*	0.056	−1.936 ***	−0.583 **			
	(0.121)	(0.455)	(0.286)			
*W × Regu × Lcte*				−0.027 ***	−0.068 *	−0.096 ***
				(0.008)	(0.035)	(0.027)
Control variables	yes	yes	yes	yes	yes	yes
Time fixed effect	yes	yes	yes	yes	yes	yes
Space fixed effect	yes	yes	yes	yes	yes	yes
*Rho*	0.080	−0.206	0.008	−0.021	−0.258	0.174
	(0.066)	(0.185)	(0.149)	(0.073)	(0.178)	(0.141)
*sigma2_e*	0.167 ***	0.016 ***	0.016 ***	0.011 ***	0.015 ***	0.015 ***
	(0.001)	(0.001)	(0.001)	(0.001)	(0.001)	(0.001)
*R-squared*	0.117	0.061	0.050	0.631	0.115	0.014
*Log-likelihood*	319.336	329.223	325.588	353.615	319.865	326.319
*Number of ID*	30	30	30	30	30	30

Note: Values in parentheses are standard errors, *** *p* < 0.01, ** *p* < 0.05, * *p* < 0.1.

## Data Availability

Data can be accessed via contacting the authors upon approval.
